# Determinants of quality of life among people with HIV and tuberculosis in an underserved area in Indonesia: a cross-sectional study

**DOI:** 10.11604/pamj.2023.46.61.41521

**Published:** 2023-10-18

**Authors:** Elfride Irawati Sianturi, Zamrotul Izzah, Khoirul Huda, Evelyn Magrid Sada, Dyah Aryani Perwitasari, Steven Yohanes Yulianus Mantiri, Elsye Gunawan

**Affiliations:** 1 Pharmacy Department, Faculty of Mathematics and Natural Sciences, University of Cenderawasih, Papua, Indonesia,; 2 Department of Clinical Pharmacy and Pharmacology, University Medical Centre Groningen, University of Groningen, Groningen, the Netherlands,; 3 Department of Pharmacy Practice, Faculty of Pharmacy, Universitas Airlangga, Surabaya, Indonesia,; 4 Faculty of Pharmacy, University of Ahmad Dahlan, Yogyakarta, Indonesia,; 5 Geophysical Engineering Study Program, Department of Physics, University of Cenderawasih, Jayapura, Indonesia

**Keywords:** WHOQOL-BREF, quality of life, tuberculosis, HIV, Indonesia

## Abstract

**Introduction:**

human immunodeficiency virus (HIV) and tuberculosis (TB) remain global health problems and impose a substantial reduction in people´s quality of life (QoL). This study aimed to assess and compare the QoL in HIV and TB patients and factors associated with QoL between those groups.

**Methods:**

a cross-sectional study was conducted at a hospital clinic in Jayapura, Indonesia, between December 2022 and March 2023. Those who were aged above 18 years, diagnosed with HIV or TB infection, have been taking HIV or TB medications for at least 3 months, and provided informed consent were eligible to participate. Patients´ QoL was measured using the Bahasa Indonesia version of a validated 26-item World Health Organization Quality of Life - Brief (WHOQOL-BREF) questionnaire.

**Results:**

a total of 365 patients with HIV and 125 with TB were included. The majority of participants were Papuan (75.9%) and aged 20 - 65 years (92.9%). More than half of the participants were female (56.5%), employed (50.8%), married (65.5%), and had family support (64.9%). Education level and social support were predictors of poor physical health in the HIV group, while ethnicity was a predictor in the TB group. Patients´ age was associated with poor psychological health in HIV, whereas sex was the associated factor in TB patients. Ethnicity was the only predictor of poor social relationships in those with TB. Whereas patients´ age was a predictor of poor environmental health in the HIV group, marital status, and education were predictors in the TB group. Finally, only social support was associated with poor general QoL in TB patients.

**Conclusion:**

tuberculosis (TB) patients had poorer QoL than those with HIV. There is a need for more awareness to support those receiving TB treatment. In addition, further research is needed to understand in more detail the determinants of patients with drug-resistant TB, TB with HIV, and drug-resistant TB-HIV, to ensure that interventions are designed to help them.

## Introduction

Quality of life (QoL) is a complex, multifaceted construct that requires multiple approaches from different theoretical angles [1]. Collaborations of scientists from various disciplines are encouraged to exploit the strengths of a collaborative effort in achieving a state of quality of life [2]. A thorough understanding of subjective well-being requires knowledge of how objective conditions influence people´s evaluations of their lives regarding the context of the culture in which they live and related diseases [3]. Similarly, a complete understanding of objective indicators and how to select them requires that we understand people´s values, and have knowledge about how objective indicators influence people´s experience of well-being [1]. Human immunodeficiency virus (HIV) and tuberculosis (TB) remain prioritized since the prevalence of both diseases has been increasing during the last five years in the Papua province, one of the underserved areas in Indonesia [4]. Papua has also struggled to manage the generalized of HIV epidemic [5], and TB as a common comorbidity for HIV will increase following the HIV prevalence. Antiretroviral therapy (ART) [6] and directly observed treatment short-course (DOTS) [7] have been widely available and accessed for free to all people with HIV or TB in Indonesia, including Papua. This commitment of the government to make treatments accessible may increase the health-related QoL of patients for both diseases. The level of QoL after patients receiving medication may be interesting to assess, and determinants associated with QoL may vary according to disease characteristics. Even though health-related QoL studies on TB [8] and HIV [9] have been performed in some urban areas in Indonesia, evidence remains limited in many contexts in this area. There is an urgent need to understand the impact of the diseases and current treatments on both TB and HIV patients. It also helps healthcare providers and other stakeholders to consider possible determinants of quality of life in HIV and TB patients. Therefore, this study examined the QoL among patients with HIV and TB as chronic diseases and their influencing factors.

## Methods

**Study design and setting:** a cross-sectional questionnaire-based study was conducted among HIV and TB patients treated at the RSUD Jayapura, a referral hospital in Jayapura-Papua, Indonesia, from December 2022 to March 2023. Jayapura, the capital of Papua province, lies in Papua Island, the most eastern of Indonesia, with a total area of 940 km^2^ and a population of 404.004 [10].

**Study population and sample size:** patients who were aged 17 years and older, both male and female and with known cases of HIV or drug-sensitive TB for at least 3 months before study enrollment were included in the study. Those who were very ill, unable to communicate, and illiterate were excluded. Purposive sampling was applied to recruit the participants. Eligible patients who attended the hospital clinic were approached individually by the researchers, who provided information about the study's background and objectives. They provided written informed consent if they agreed to participate. Participation in the study was voluntary, and the participants may withdraw at any time during the study. The sample size was determined using a single population proportion formula with a 95% confidence interval and a 5% margin of error [11]. Based on the total population of approximately 2000 patients with HIV or TB visiting the hospital clinic, the minimum sample size was 323. Finally, with the addition of 10% withdrawal, at least 355 patients were recruited.

**Data collection:** participants´ sociodemographic characteristics, including age (in years), sex (female or male), marital status (single, married, or divorced/widowed), education level (primary, secondary, or tertiary), occupation (unemployed, retired, or employed), monthly income, region of residence, and social support available (spouse, family, or none) were collected. Patients´ age was also categorized into 17 - 20 years (young adult), 20 - 65 years (adult), and above 65 years (older adult) [12]. Clinical information on current HIV or TB treatment was also recorded. The primary outcome measure was patients´ QoL, assessed by using the Bahasa Indonesia version of the validated 26-item of World Health Organization Quality of Life - Brief (WHOQOL-BREF) questionnaire.

The WHOQOL-BREF questionnaire is a 26-item, self-directed, non-specific questionnaire that is a short form of the WHOQOL-100 scale and publicly available [13]. Standardized instructions to the interviewers helped in the acquisition of the WHOQOL-BREF administration [14]. The questionnaire examines four domains of perceived health-related QoL, namely physical health, psychological, social relationship, and environmental health in addition to a general QoL. The first two items in the scale ask about an individual's overall perception of QoL and an individual's overall perception of their health. The other items represent a person's perception of their QoL in each of the four domains. The time span covers the last two weeks. Each item of the questionnaire is measured on a 5-point Likert scale, ranging from 1 to 5 with varying scale responses and higher values representing higher QoL. The domain scores were calculated by multiplying the mean score of each domain by four according to WHOQOL-BREF scoring manual. The QoL scores of each domain were dichotomized into “good” and “poor” with a score lower than 60 defined as “poor” [15].

**Data analysis:** descriptive statistics were used to describe the sociodemographic and disease characteristics of the participants. The Kolmogorov-Smirnov test and a Q-Q plot were used to evaluate whether the continuous variables had a normal distribution. Data were presented in frequencies and percentages for categorical variables and means ± standard deviations (SDs) or medians ± interquartile ranges (IQRs) for continuous variables. Furthermore, the associations between the QoL domains and current HIV or TB treatment were tested using the Mann-Whitney test. Binary logistic regression analysis was performed to identify factors associated with each QoL domain. The final multivariable models comprised covariates which were selected using a backward stepwise approach, and the final model retained variables with p-value <0.25. The results were stratified by current HIV or TB treatment and reported as odds ratios (ORs) with a 95% confidence interval (CI). All analyses were done using two-tailed tests at a significance level of 0.05. The statistical analyses were performed using the Statistical Program for Social Sciences (SPSS) version 24.0 for Windows.

**Ethical consideration:** in this study, participation was voluntary. The study protocol has been reviewed and approved by the ethics committee at Muhammadiyah Magelang University 029/KEPK-FIKES/II.3.AU/F/2023). All participants were required to provide written informed consent prior to data collection.

**Funding:** the authors (Elfride Irawati Sianturi and Steven Yohanes Yulianus Mantiri) received a grant from DIKTI (016/E5/PG.02.00.PL/2023)

## Results

**Sample characteristics:** in total, 490 patients were included in the study. Of them, 365 patients (74.5%) were undergoing HIV treatment, and 125 (25.5%) patients were currently treated for TB infection (Table 1). The majority of participants were aged 20 - 65 years (92.9%) at enrollment, female (56.5%), and Papuan (75.9%). More than half of the participants were employed (50.8%), married (65.5%), and had family support (64.9%).

**Table 1:** patient characteristics by current HIV or TB treatment

Variable	Frequency (%)		
	Total (N=490)	HIV treatment (N=365)	TB treatment (N=125)
**Age (years)**			
12-20 (young adult)	27 (5.5)	9 (2.5)	18 (14.4)
20-65 (adult)	455 (92.9)	354 (97.0)	101 (80.8)
>65 (older adult)	8 (1.6)	2 (0.5)	6 (4.8)
Age (years)	34.85 ±11.4	35.11 ±10.6	34.10±13.3
**Sex**			
Female	277 (56.5)	210 (57.5)	67 (53.6)
Male	213 (43.5)	155 (42.5)	58 (46.4)
**Ethnic**			
Papuan	372 (75.9)	272 (74.5)	100 (80.0)
Non-Papuan	118 (24.1)	93 (25.5)	25 (20.0)
**Marital status**			
Single	155 (31.6)	115 (31.5)	40 (32.0)
Married	323 (65.9)	250 (68.5)	73 (58.4)
Divorced/widowed	12 (2.4)	0 (0)	12 (9.6)
**Education level**			
Primary	36 (7.3)	25 (6.8)	11 (8.8)
Secondary	365 (74.5)	275 (75.3)	90 (72.0)
Tertiary	89 (18.2)	65 (17.8)	24 (19.2)
**Occupation**			
Unemployed	235 (48.0)	169 (46.3)	66 (52.8)
Retired	6 (1.2)	1 (0.3)	5 (4.0)
Employed	249 (50.8)	195 (53.4)	54 (43.2)
**Social support**			
None	19 (3.9)	19 (5.2)	0 (0)
Spouse	153 (31.2)	90 (24.7)	63 (50.4)
Family	318 (64.9)	256 (70.1)	62 (49.6)

HIV, human immunodeficiency virus; TB, tuberculosis

**All domains of quality of life:** differences between HIV and TB treatment groups in the four domains of QoL were described in Table 2. An analysis of mean scores across individual measures showed that those with HIV had higher physical health, social relationships, environmental health, and general QoL than TB patients. The exception was seen in the domain of psychological health, where those with TB treatment had higher than those with HIV. Significant differences were found in most part of the domains except for environmental health, suggesting that TB patients had poorer QoL than those with HIV (p<0.001). In addition, the general QoL was significantly higher in the HIV group than in the TB group (p<0.001).

**Table 2:** domains of health-related quality of life by current HIV or TB treatment

Domain	Mean (SD)		Mann-Whitney U test p-value
	HIV-treatment	TB treatment	
Physical health	67.06±9.19	57.03±13.57	<0.001^a^
Psychological health	64.10±8.89	77.23 ±13.66	<0.001^a^
Social relationship	66.59±10.67	61.13±17.03	<0.001^a^
Environmental Health	72.54±9.30	71.97 ±12.68	0.89
The general quality of life	86.33±14.15	70.20±30.52	<0.001^a^

a Statistically significant at a p-value less than 0.05 HIV, human immunodeficiency virus; SD, Standard Deviation; TB, tuberculosis

**Physical health:** whereas education level and social support were factors associated with poor physical health of HIV patients, ethnicity was associated with poor physical health of TB patients in the final multivariable models (Table 3). In Table 3, patients with HIV who had secondary education were less likely to have poor physical health (adjusted OR [aOR]: 0.21, 95%CI: 0.08 - 0.52) relative to those with primary education. HIV patients who had spouse and family support also decreased the odds of having poor physical health (aOR: 0.19, 95%CI: 0.05 - 0.66) and aOR: 0.34, 95%CI: 0.12 - 0.98, respectively). In the TB group, being non-Papuan lowered the odds of having poor physical health (aOR: 0.32, 95%CI: 0.12 - 0.88) than Papuans.

**Table 3:** factors associated with poor physical health by current HIV or TB treatment

HIV treatment							TB Treatment					
Variable	Physical health		Odds ratio^b^ (95% CI)	p-value	Adjusted odds^c^ ratio (95% CI)	p-value	Physical health		Odds ratio^b^ (95% CI)	p-value	Adjusted oddsc ratio (95% CI)	p-value
	Good n (%)	Poor^a^ n (%)					Good n (%)	Poor^a^ n (%)				
**Age (years)**	314 (86.0)	51 (14.0)	1.00 (0.97 – 1.03)	0.990			55 (44.0)	70 (56.0)	1.01 (0.98 – 1.04)	0.540		
**Age category (years)**												
12-20 (young adult)	6 (1.9)	3 (5.9)	1.00				9 (16.4)	9 (12.9)	1.00			
20-65 (adult)	307 (97.8)	47 (92.2)	0.31 (0.07 – 1.27)	0.100			43 (78.2)	58 (82.9)	1.35 (0.49 – 3.68)	0.560		
>65 (older adult)	1 (0.3)	1 (2.0)	2.00 (0.09 – 44.35)	0.660			3 (5.5)	3 (4.3)	1.00 (0.16 – 6.35)	1.000		
**Sex**												
Female	186 (59.2)	24 (47.1)	1.00		1.00		30 (54.5)	37 (52.9)	1.00			
Male	128 (40.8)	27 (52.9)	1.64 (0.90 – 2.96)	0.110	1.69 (0.90 – 3.14)	0.100	25 (45.5)	33 (47.1)	1.07 (0.53 – 2.17)	0.850		
**Ethnic**												
Papuan	237 (75.5)	35 (68.6)	1.00				40 (72.7)	60 (85.7)	1.00		1.00	
Non-Papuan	77 (24.5)	16 (31.4)	1.41 (0.74 – 2.68)	0.300			15 (27.3)	10 (14.3)	0.44 (0.18 – 1.09)	0.080	0.32 (0.12 – 0.88)	0.027^d^
**Marital status**												
Single	99 (31.5)	16 (31.4)	1.00				19 (34.5)	21 (30.0)	1.00			
Married	215 (68.5)	35 (68.6)	1.01 (0.53 – 1.91)	0.980			29 (52.7)	44 (62.9)	1.37 (0.63 – 2.99)	0.430		
Divorced/widowed	0	0	N/A				7 (12.7)	5 (7.1)	0.65 (0.18 – 2.38)	0.510		
**Education level**												
Primary	16 (5.1)	9 (17.6)	1.00		1.00		2 (3.6)	9 (12.9)	1.00		1.00	
Secondary	244 (77.7)	31 (60.8)	0.23 (0.09 – 0.55)	0.001	0.21 (0.08 – 0.52)	<0.001d	47 (85.5)	43 (61.4)	0.20 (0.04 – 0.99)	0.049	0.21 (0.04 – 1.06)	0.06
Tertiary	54 (17.2)	11 (21.6)	0.36 (0.13 – 1.03)	0.060	0.34 (0.12 – 1.01)	0.053	6 (10.9)	18 (25.7)	0.67 (0.11 – 3.99)	0.660	0.96 (0.15 – 6.16)	0.97
**Occupation**												
Unemployed	149 (47.5)	20 (39.2)	1.00				31 (56.4)	35 (50.0)	1.00			
Retired	1 (0.3)	0	N/A				2 (3.6)	3 (4.3)	1.33 (0.21 – 8.48)	0.760		
Employed	164 (52.2)	31 (60.8)	1.41 (0.77 – 2.58)	0.270			22 (40.0)	32 (45.7)	1.29 (0.62 – 2.66)	0.490		
**Social support**												
None	13 (4.1)	6 (11.8)	1.00		1.00		0	0	N/A			
Spouse	83 (26.4)	7 (13.7)	0.18 (0.05 – 0.63)	0.007	0.19 (0.05 – 0.66)	0.009d	29 (52.7)	34 (48.6)	1.00			
Family	218 (69.4)	38 (74.5)	0.38 (0.14 – 1.06)	0.060	0.34 (0.12 – 0.98)	0.047d	26 (47.3)	36 (51.4)	1.18 (0.58 – 2.39)	0.650		

a Poor physical health was defined by a score of physical health less than 60.0 ^b^Odds ratio derived from the univariate logistic regression analysis ^c^Adjusted odds ratio of predictors in the final multivariable logistic regression model ^ad^Statistically significant at a p-value less than 0.05 CI, confidence interval; HIV, human immunodeficiency virus; N/A, not available; TB, tuberculosis

**Psychological health:** factors associated with poor psychological health by treatment HIV and TB treatment groups are shown in Table 4. In the HIV group, each additional yearly increase in age decreased the odds of having poor psychological health (aOR: 0.96, 95%CI: 0.94 - 0.99). Being male than female was less likely to have poor psychological health in TB patients (aOR: 0.15, 95%CI: 0.03 - 0.82).

**Table 4:** factors associated with poor psychological health by current HIV or TB treatment

HIV treatment							TB Treatment					
Variable	Psychological health		Odds ratio^b^ (95% CI)	p-value	Adjusted odds ratio^c^ (95% CI)	p-value	Variable		Odds ratio^b^ (95% CI)	p-value	Adjusted odds^c^ ratio (95% CI)	p-value
	Good n (%)	Poor^a^ n (%)					Good n (%)	Poor^a^ n (%)				
Age (years)	205 (56.2)	160 (43.8)	0.98 (0.96 – 1.00)	0.090	0.96 (0.94 – 0.99)	0.002^d^	112 (89.6)	13 (10.4)	1.01 (0.97 – 1.05)	0.760	1.06 (0.99 – 1.15)	0.11
**Age category (years)**												
12-20 (young adult)	5 (2.4)	4 (2.5)	1.00		1.00		17 (15.2)	1 (7.7)	1.00			
20-65 (adult)	200 (97.6)	154 (96.3)	0.96 (0.25 – 3.65)	0.960	1.16 (0.29 – 4.66)	0.840	89 (79.5)	12 (92.3)	2.29 (0.28 – 18.81)	0.440		
>65 (older adult)	0	2 (1.3)	N/A		N/A		6 (5.4)	0	N/A			
**Sex**												
Female	122 (59.5)	88 (55.0)	1.00				57 (50.9)	10 (76.9)	1.00		1.00	
Male	83 (40.5)	72 (45.0)	1.20 (0.79 – 1.83)	0.390			55 (49.1)	3 (23.1)	0.31 (0.08 – 1.19)	0.090	0.15 (0.03 – 0.82)	0.028^d^
**Ethnic**												
Papuan	152 (74.1)	120 (75.0)	1.00				87 (77.7)	13 (100.0)	1.00			
Non-Papuan	53 (25.9)	40 (25.0)	0.96 (0.59 – 1.54)	0.850			25 (22.3)	0	N/A			
**Marital status**												
Single	66 (32.2)	49 (30.6)	1.00		1.00		36 (32.1)	4 (30.8)	1.00		1.00	
Married	139 (67.8)	111 (69.4)	1.08 (0.69 – 1.68)	0.750	1.47 (0.88 – 2.46)	0.140	64 (57.1)	9 (69.2)	1.27 (0.36 – 4.40)	0.710	0.70 (0.13 – 3.83)	0.6800
Divorced/widowed	0	0	N/A		N/A		12 (10.7)	0	N/A		N/A	
**Education level**												
Primary	11 (5.4)	14 (8.8)	1.00				9 (8.0)	2 (15.4)	1.00		1.00	
Secondary	158 (77.1)	117 (73.1)	0.58 (0.26 – 1.33)	0.200			83 (74.1)	7 (53.8)	0.38 (0.07 – 2.11)	0.270	0.47 (0.07 – 3.27)	0.450
Tertiary	36 (17.6)	29 (18.1)	0.63 (0.25 – 1.60)	0.330			20 (17.9)	4 (30.8)	0.90 (0.14 – 5.84)	0.910	2.34 (0.26 – 21.47)	0.450
**Occupation**												
Unemployed	101 (49.3)	68 (42.5)	1.00		1.00		59 (52.7)	7 (53.8)	1.00			
Retired	0	1 (0.6)	N/A		N/A		5 (4.5)	0	N/A			
Employed	104 (50.7)	91 (56.9)	1.30 (0.86 – 1.97)	0.220	1.47 (0.95 – 2.28)	0.080	48 (42.9)	6 (46.2)	1.05 (0.33 – 3.34)	0.930		
**Social support**												
None	13 (6.3)	6 (3.8)	1.00				0	0	N/A		N/A	
Spouse	52 (25.4)	38 (23.8)	1.58 (0.55 – 4.54)	0.390			59 (52.7)	4 (30.8)	1.00		1.00	
Family	140 (68.3)	116 (72.5)	1.80 (0.66 – 4.87)	0.250			53 (47.3)	9 (69.2)	2.51 (0.73 – 8.61)	0.150	3.17 (0.78 – 12.98)	0.110

a Poor psychological health was defined by a score of physical health less than 60.0. ^b^Odds ratio derived from the univariate logistic regression analysis. ^c^Adjusted odds ratio of predictors in the final multivariable logistic regression model. ^d^Statistically significant at p-value less than 0.05. CI, confidence interval; HIV, human immunodeficiency virus; N/A, not available; TB, tuberculosis.

**Social relationship:** Table 5 shows no variable was significantly associated with poor social relationships among patients with HIV. However, being non-Papuan was less likely to have poor social health in TB patients (aOR: 0.35, 95%CI: 0.12 - 0.98) relative to Papuans.

**Table 5:** factors associated with poor social health by current HIV or TB treatment

HIV treatment							TB Treatment					
Variable	Social health		Odds ratio^b^ (95% CI)	p-value	Adjusted odds ratio^c^ (95% CI)	p-value	Variable		Odds ratio^b^ (95% CI)	p-value	Adjusted odds^c^ ratio (95% CI)	p-value
	Good n (%)	Poor^a^ n (%)					Good n (%)	Poor^a^ n (%)				
**Age (years)**	213 (58.4)	152 (41.6)	1.01 (0.99 – 1.03)	0.500			68 (54.4)	57 (45.6)	0.97 (0.95 – 1.00)	0.070	0.97 (0.94 – 1.00)	0.060
**Age category (years)**												
12-20 (young adult)	8 (3.8)	1 (0.7)	1.00		1.00		7 (10.3)	11 (19.3)	1.00			
20-65 (adult)	205 (96.2)	149 (98.0)	5.82 (0.72 – 46.99)	0.100	4.47 (0.54 – 37.11)	0.170	57 (83.8)	44 (77.2)	0.49 (0.18 – 1.37)	0.170		
>65 (older adult)	0	2 (1.3)	N/A		N/A		4 (5.9)	2 (3.5)	0.32 (0.05 – 2.22)	0.250		
**Sex**												
Female	121 (56.8)	89 (58.6)	1.00				37 (54.4)	30 (52.6)	1.00			
Male	92 (43.2)	63 (41.4)	0.93 (0.61 – 1.42)	0.740			31 (45.6)	27 (47.4)	1.07 (0.53 – 2.18)	0.840		
**Ethnic**												
Papuan	159 (74.6)	113 (74.3)	1.00				50 (73.5)	50 (87.7)	1.00		1.00	
Non-Papuan	54 (25.4)	39 (25.7)	1.02 (0.63 – 1.64)	0.950			18 (26.5)	7 (12.3)	0.39 (0.15 – 1.01)	0.050	**0.35 (0.12 – 0.98)**	**0.046d**
**Marital status**												
Single	76 (36.7)	39 (25.7)	1.00		1.00		21 (30.9)	19 (33.3)	1.00			
Married	137 (64.3)	113 (74.3)	1.61 (1.01 – 2.55)	0.040	1.45 (0.91 – 2.32)	0.120	39 (57.4)	34 (59.6)	0.96 (0.45 – 2.09)	0.930		
Divorced/widowed	0	0	N/A		N/A		8 (11.8)	4 (7.0)	0.55 (0.14 – 2.13)	0.390		
**Education level**												
Primary	14 (6.6)	11 (7.2)	1.00				6 (8.8)	5 (8.8)	1.00		1.00	
Secondary	160 (75.1)	115 (75.7)	0.92 (0.40 – 2.09)	0.830			52 (76.5)	38 (66.7)	0.88 (0.25 – 3.09)	0.840	0.61 (0.15 – 2.46)	0.490
Tertiary	39 (18.3)	26 (17.1)	0.85 (0.33 – 2.16)	0.730			10 (14.7)	14 (24.6)	1.68 (0.40 – 7.08)	0.480	1.83 (0.38 – 8.84)	0.450
**Occupation**												
Unemployed	103 (48.4)	66 (43.4)	1.00				33 (48.5)	33 (57.9)	1.00			
Retired	0	1 (0.7)	N/A				3 (4.4)	2 (3.5)	0.67 (0.10 – 4.25)	0.670		
Employed	110 (51.6)	85 (55.9)	1.21 (0.79 – 1.83)	0.380			32 (47.1)	22 (38.6)	0.69 (0.33 – 1.42)	0.310		
**Social support**												
None	12 (5.6)	7 (4.6)	1.00				0	0	N/A			
Spouse	55 (25.8)	35 (23.0)	1.09 (0.39 – 3.04)	0.870			34 (50.0)	29 (50.9)	1.00			
Family	146 (68.5)	110 (72.4)	1.29 (0.49 – 3.39)	0.600			34 (50.0)	28 (49.1)	0.97 (0.48 – 1.95)	0.920		

a Poor social health was defined by a score of physical health less than 60.0. ^b^Odds ratio derived from the univariate logistic regression analysis. ^c^Adjusted odds ratio of predictors in the final multivariable logistic regression model. ^d^Statistically significant at a p-value less than 0.05. CI, confidence interval; HIV, human immunodeficiency virus; N/A, not available; TB, tuberculosis.

**Environmental health:** age was associated with poor environmental health in the HIV group (Table 6). An increase of one year in age of HIV patients lowered the odds of having poor environmental health (aOR: 0.96, 95%CI: 092 - 0.99), but being an older adult was 88 times more likely to have poor environmental health (aOR: 87.91, 95%CI: 1.46 - 5290.43) relative to the other age groups. In Table 6, the TB group, marital status, and level of education predicted poor environmental health. Being married increased the odds of having poor environmental health by five-fold (aOR: 5.35, 95% I: 1.26 - 22.65). TB patients who had secondary education were less likely to have poor environmental health (aOR: 0.11, 95%CI: 0.02 - 0.67).

**Table 6:** factors associated with poor environmental health by current HIV or TB treatment

HIV treatment							TB Treatment					
Variable	Environmental health		Odds ratio^b^ (95% CI)	p-value	Adjusted odds ratio^c^ (95% CI)	p-value	Environmental health		Odds ratio^b^ (95% CI)	p-value	Adjusted odds^c^ ratio(95% CI)	p-value
	Good n (%)	Poor^a^ n (%)					Good n (%)	Poor^a^ n (%)				
**Age (years)**	328 (89.9)	37 (10.1)	0.97 (0.94 – 1.01)	0.12	**0.96 (0.92 – 0.99)**	**0.032d**	100 (80.0)	25 (20.0)	0.98 (0.95 – 1.02)	0.30	0.94 (0.87 – 1.01)	0.07
**Age category (years)**												
12-20 (young adult)	8 (2.4)	1 (2.7)	1.00		1.00		16 (16.0)	2 (8.0)	1.00		1.00	
20-65 (adult)	319 (97.3)	35 (94.6)	0.88 (0.11 – 7.23)	0.90	1.63 (0.19 – 14.19)	0.66	78 (78.0)	23 (92.0)	2.36 (0.51 – 11.02)	0.28	2.30 (0.36 – 14.74)	0.38
>65 (older adult)	1 (0.3)	1 (2.7)	8.00 (0.25 – 255.75)	0.24	**87.91 (1.46 – 5290.43)**	**0.032^d^**	6 (6.0)	0	N/A		N/A	
**Sex**												
Female	187 (57.0)	23 (62.2)	1.00				50 (50.0)	17 (68.0)	1.00			
Male	141 (43.0)	14 (37.8)	0.81 (0.40 – 1.63)	0.55			50 (50.0)	8 (32.0)	0.47 (0.19 – 1.19)	0.11		
**Ethnic**												
Papuan	244 (74.4)	28 (75.7)	1.00				78 (78.0)	22 (88.0)	1.00		1.00	
Non-Papuan	84 (25.6)	9 (24.3)	0.93 (0.42 – 2.06)	0.87			22 (22.0)	3 (12.0)	0.48 (0.13 – 1.77)	0.27	0.42 (0.10 – 1.77)	0.24
**Marital status**												
Single	102 (31.1)	13 (35.1)	1.00				34 (34.0)	6 (24.0)	1.00		1.00	
Married	226 (68.9)	24 (64.9)	0.83 (0.41 – 1.70)	0.62			56 (56.0)	17 (68.0)	1.72 (0.62 – 4.79)	0.30	**5.35 (1.26 – 22.65)**	**0.023d**
Divorced/widowed	0	0	N/A				10 (10.0)	2 (8.0)	1.13 (0.20 – 6.51)	0.89	4.70 (0.29 – 77.37)	0.28
**Education level**												
Primary	23 (7.0)	2 (5.4)	1.00				7 (7.0)	4 (16.0)	1.00		1.00	
Secondary	247 (75.3)	28 (75.7)	1.30 (0.29 – 5.82)	0.73			77 (77.0)	13 (52.0)	0.30 (0.08 – 1.15)	0.08	**0.11 (0.02 – 0.67)**	**0.02^d^**
Tertiary	58 (17.7)	7 (18.9)	1.39 (0.27 – 7.18)	0.70			16 (16.0)	8 (32.0)	0.88 (0.20 – 3.90)	0.86	0.57 (0.08 – 3.82)	0.56
**Occupation**												
Unemployed	151 (46.0)	18 (48.6)	1.00				51 (51.0)	15 (60.0)	1.00		1.00	
Retired	1 (0.3)	0	N/A				4 (4.0)	1 (4.0)	0.85 (0.09 – 8.19)	0.89	11.42 (0.13 – 1009.22)	0.29
Employed	176 (53.7)	19 (51.4)	0.91 (0.46 – 1.79)	0.78			45 (45.0)	9 (36.0)	0.68 (0.27 – 1.70)	0.41	0.31 (0.09 – 1.12)	0.07
**Social support**												
None	18 (5.5)	1 (2.7)	1.00				0	0	N/A			
Spouse	82 (25.0)	8 (21.6)	1.76 (0.21 – 14.93)	0.61			49 (49.0)	14 (56.0)	1.00			
Family	228 (69.5)	28 (75.7)	2.21 (0.28 – 17.20)	0.45			51 (51.0)	11 (44.0)	0.76 (0.31 – 1.82)	0.53		

a Poor environmental health was defined by a score of physical health less than 60.0 ^b^ Odds ratio derived from the univariate logistic regression analysis ^c^ Adjusted odds ratio of predictors in the final multivariable logistic regression model ^d^Statistically significant at p-value less than 0.05 CI, confidence interval; HIV, human immunodeficiency virus; N/A, not available; TB, tuberculosis

**General quality of life:** after adjusting all independent variables among HIV patients, no independent variable was associated with poor general QoL (Table 7). Only social support predicted poor general QoL in TB patients. Those who had family support were twice as likely to have poor general QoL (aOR: 2.40, 95%CI: 1.06 - 5.45) relative to those supported by their spouses.

**Table 7:** factors associated with poor general quality of life by current HIV or TB treatment

HIV treatment							TB Treatment					
Variable	General Quality of Life		Odds ratio^b^ (95%CI)	p-value	Adjusted odds ratio^c^ (95%CI)	p-value	General Quality of Life		Odds ratio^b^ (95%CI)	p-value	Adjusted odds^c^ ratio (95%CI)	p-value
	Good n (%)	Poor^a^ n (%)					Good n (%)	Poor^a^ n(%)				
**Age (years)**	353 (96.7)	12 (3.3)	0.96 (0.90 – 1.03)	0.230	0.96 (0.90 – 1.03)	0.220	84 (67.2)	41 (32.8)	0.98 (0.95 – 1.01)	0.140		
**Age category (years)**												
12-20 (young adult)	8 (2.3)	1 (8.3)	1.00				11 (13.1)	7 (17.1)	1.00		1.00	
20-65 (adult)	343 (97.2)	11 (91.7)	0.26 (0.03 – 2.23)	0.220			68 (81.0)	33 (80.5)	0.76 (0.27 – 2.15)	0.610	0.72 (0.20 – 2.60)	0.610
>65 (older adult)	2 (0.6)	0	N/A				5 (6.0)	1 (2.4)	0.31 (0.03 – 3.29)	0.330	N/A	
**Sex**												
Female	205 (58.1)	5 (41.7)	1.00				44 (52.4)	23 (56.1)	1.00		1.00	
Male	148 (41.9)	7 (58.3)	1.94 (0.60 – 6.23)	0.270			40 (47.6)	18 (43.9)	0.86 (0.41 – 1.82)	0.700	0.45 (0.17 – 1.18)	0.100
**Ethnic**												
Papuan	265 (75.1)	7 (58.3)	1.00		1.00		65 (77.4)	35 (85.4)	1.00			
Non-Papuan	88 (24.9)	5 (41.7)	2.15 (0.67 – 6.95)	0.200	2.20 (0.68 – 7.12)	0.190	19 (22.6)	6 (14.6)	0.59 (0.22 – 1.60)	0.300		
**Marital status**												
Single	111 (31.4)	4 (33.3)	1.00				24 (28.6)	16 (39.0)	1.00		1.00	
Married	242 (68.6)	8 (66.7)	0.92 (0.27 – 3.11)	0.890			48 (57.1)	25 (61.0)	0.78 (0.35 – 1.73)	0.540	0.55 (0.19 – 1.60)	0.270
Divorced/widowed	0	0	N/A				12 (14.3)	0	N/A		N/A	
**Education level**												
Primary	25 (7.1)	0	N/A				8 (9.5)	3 (7.3)	0.79 (0.20 – 3.19)	0.740		
Secondary	265 (75.1)	10 (83.3)	1.00				61 (72.6)	29 (70.7)	1.00			
Tertiary	63 (17.8)	2 (16.7)	0.84 (0.18 – 3.94)	0.830			15 (17.9)	9 (22.0)	1.26 (0.49 – 3.22)	0.630		
**Occupation**												
Unemployed	164 (46.5)	5 (41.7)	1.00				44 (52.4)	22 (53.7)	1.00		1.00	
Retired	1 (0.3)	0	N/A				5 (6.0)	0	N/A		N/A	
Employed	188 (53.3)	7 (58.3)	1.22 (0.38 – 3.92)	0.740			35 (41.7)	19 (46.3)	1.09 (0.51 – 2.32)	0.830	2.46 (0.77 – 7.86)	0.130
**Social support**												
None	17 (4.8)	2 (16.7)	1.00				0	0	N/A		N/A	
Spouse	88 (24.9)	2 (16.7)	0.19 (0.03 – 1.47)	0.110			48 (57.1)	15 (36.6)	1.00		1.00	
Family	248 (70.3)	8 (66.7)	0.27 (0.05 – 1.39)	0.120			36 (42.9)	26 (63.4)	2.31 (1.07 – 4.98)	0.030	2.40 (1.06 – 5.45)	0.036^d^

a Poor **General Quality of Life** was defined by the score of physical health less than 60.0 ^b^Odds ratio derived from the univariate logistic regression analysis ^c^Adjusted odds ratio of predictors in the final multivariable logistic regression model ^d^Statistically significant at a p-value less than 0.05 CI, confidence interval; HIV, human immunodeficiency virus; N/A, not available; TB, tuberculosis

## Discussion

The study findings reveal that TB patients had poorer QoL than those with HIV. Education level and social support predicted poor physical health in the HIV group, while ethnicity was a predictor in the TB group. The age of a patient was associated with poor psychological health among HIV patients, whereas patients´ sex was the associated factor in TB patients. Furthermore, ethnicity was the only predictor of poor social relationships in those with TB. Whereas patients´ age predicted poor environmental health in the HIV group, marital status and education were predictors in the TB group. Finally, only social support was associated with poor general QoL in TB patients.

The present study provides consistent results with the previous reports. Those on HIV treatment [16,17]. Despite this, QoL in people with HIV is affected not only by the virus but also by the stigma after diagnosis. The complexity of HIV treatment is poorly explained in terms of social relationships and general QoL. It is likely that there are other variables that may have a greater impact on these areas than those used in this study. Numerous studies have shown that stigma has a significant impact on many aspects of the lives of people living with HIV, including their QoL [18-20]. Surprisingly, there is no significant determinant of poor social relationships among people with HIV. A possible explanation could be that there are other factors related to social health in addition to the existing determinants. Thus, future studies addressing more magnitude factors are warranted. Influencing factors such as fear of HIV disclosure and being stigmatized by others may have the greatest impact on the social health determinant [21,22].

Social support is shown to affect the HIV patients´ physical health and the TB patients´ general QoL. Individuals with symptomatic HIV disease might be vulnerable to visiting hospitals to collect antiretroviral drugs. Furthermore, their fear and self-acceptance will be easier to get through when social support is present, particularly when they are too sick [22]. This finding is inconsistent with a previous study that showed social support from family corresponded with better QoL among TB patients [23]. However, another study reported that social support was more likely to have poor QoL since those with TB considered emotional support as a burden to them and seemed to remind them that their disease could infect others [24]. Feelings of avoidance are common in the early course of TB medication when they tend to cause distance. These groups often seem to feel socially isolated and disconnected from friends and family, which is partly due to a lack of social-emotional skills. Contrary to previous research, people with TB are more likely to feel alone when taking medication [24].

Ethnicity became one of the predictors of poor physical health and social relationships among those with TB. This finding can occur to those with TB since it is common to find Papuans were under diagnosis and under treatment. This study showed that non-Papuans tended to have better physical health than Papuans, because non-Papuans may take coughing seriously as one of the symptoms of TB [25] and they preferred to seek help from healthcare facilities rather than using self-medication to make the cough go away. Compared to non-Papuans, Papuans may be delayed in getting diagnosed. Although the complex or residual disease may also appear to develop HIV, people living with TB have never expected to experience severe TB symptoms similar to those with HIV. Community communication and awareness of TB may be lower than HIV [26]. Being non-Papuan with TB will be more resilient to cope with disability compared to Papuans. Similar to physical health, there is an assumption that Papuans must reduce their communal activities to prevent infection to others. This may also be due to a lack of social contact and having few people to interact with on a regular basis. Even the sense of social belonging becomes distant.

Those who have at least secondary education were less likely to have poor physical health in the HIV group and poor environmental health in the TB group. Education could reduce the risk of negative reactions from others. It shows that educating people can help them understand how taking medication regularly leads to a good quality of life. Moreover, by having an education, those with TB have more exposure to how to be in good physical health compared to people who are not educated. Inadequate knowledge can lead to fear of transmission of these diseases. This can be more difficult for those who have been married, and it leads to less intimacy with their spouse and children. It is thought that age is related to people's ability to engage in proactive coping, where they are able to focus on the positive rather than just the fear of their illness [27]. Getting older makes people more resilient to achieve happiness and life balance than the effect of HIV on neuro-physiological [28]. Moreover, perhaps the issue of transmission is difficult to exclude, and it might add feelings of loneliness, especially among younger people with HIV since they will face the reality of their future seeming to disappear. Meanwhile, for psychological health, male sex influences psychological health among those with TB.

Being married was a predictor of poor environmental health, and it may be more likely to be alone among those with TB. It makes sense that these groups would automatically make distance since the diseases were diagnosed to them. It may be related to how people become infected. Although these barriers are common to people with HIV, this study found that those on TB treatment were more vulnerable than those with HIV. Of note, TB can spread from person to person through the air nearby, and people who breathe in the TB bacteria can easily get TB. This study has included a relatively large sample of thoroughly diagnosed patients with TB and HIV characteristic measures, as well as a relatively large number of indigenous Papuans and non-Papuans. However, the cross-sectional nature of the self-reported study precludes cause-and-effect statements, as well as the potential for reverse causality, and makes it difficult to establish causality. However, these cross-sectional data allow us to better understand which important patient factors are associated with health-related QoL for future studies.

## Conclusion

tuberculosis patients had poorer QoL than those with HIV in the study population. Age, sex, ethnicity, education level, marital status, and social support were shown to have an association with the different health-related QoL domains. There is a need for more awareness to support those receiving TB treatment. In addition, further research is needed to understand in more detail the determinants of patients with drug-resistant TB, TB with HIV, and drug-resistant TB-HIV, to ensure that interventions are designed to help them.

### What is known about this topic

- *Quality of life (QoL) is a complex, multifaceted construct that requires multiple approaches from different theoretical angles;*
- *The level of QoL after patients receive medication may be interesting to assess and determinants associated with QoL may vary to disease characteristics;*
- *Those on HIV treatment were generally more likely to have better QoL than those on TB treatment*.

### What this study adds

- *Those on HIV treatment were generally more likely to have better QoL than those on TB treatment;*
- *Feelings of avoidance are common in the early course of TB medication, and they often seem to feel socially isolated and disconnected from friends and family, which is partly due to a lack of social-emotional skills*.
